# Target-Specific Fluorescence-Mediated Tomography for Non-Invasive and Dynamic Assessment of Early Neutrophil Infiltration in Murine Experimental Colitis

**DOI:** 10.3390/cells8111328

**Published:** 2019-10-28

**Authors:** Tobias M. Nowacki, Philipp Lenz, Dominik Bettenworth, Markus Brückner, Arne Bokemeyer, Phil R. Tepasse, Anne Helfen, Moritz Wildgruber, Michel Eisenblätter

**Affiliations:** 1 Department of Medicine B, Gastroenterology and Hepatology, University Hospital Münster, D-48149 Münster, Germany; 2 Institute of Palliative Care, University of Münster, D-48149 Münster, Germany; 3 Translational Research Imaging Center, Department of Clinical Radiology, University Hospital Münster, D-48149 Münster, Germany; anne.helfen@ukmuenster.de (A.H.);; 4 Department of Diagnostic and Interventional Radiology, University Medical Center Freiburg, D-79106 Freiburg, Germany

**Keywords:** in vivo imaging, diagnostic imaging, experimental colitis, dextran sulfate sodium colitis, inflammatory bowel disease, fluorescence imaging, murine endoscopy

## Abstract

The role of neutrophils in the pathogenesis of inflammatory bowel disease (IBD) is still only incompletely understood. Here, we evaluated target-specific fluorescence-mediated tomography (FMT) for visualization of neutrophil infiltration in murine experimental DSS-induced colitis. Colitis was assessed using clinical, endoscopic, and histopathological parameters. Intestinal neutrophil infiltration was determined at day 0, 4, and 10 by targeted FMT after injection of a neutrophil-specific fluorescence-labelled monoclonal antibody (Gr-1). Complementary, immunofluorescence tissue sections with Gr-1 and ELISA-based assessment of tissue myeloperoxidase (MPO) served as the gold standard for the quantification of neutrophil infiltration. Colitic animals showed decreasing body weight, presence of fecal occult blood, and endoscopic signs of inflammation. FMT revealed a significantly increased level of fluorescence only four days after colitis induction as compared to pre-experimental conditions (pmol tracer 73.2 ± 18.1 versus 738.6 ± 80.7; *p* < 0.05), while neither body weight nor endoscopic assessment showed significant changes at this early time. Confirmatory, post-mortem immunofluorescence studies and measurements of tissue MPO confirmed the presence of increased neutrophil infiltration in colitic mice compared to controls. Concluding, Gr-1 targeted FMT can detect early colonic infiltration of neutrophils in experimental colitis even before clinical symptoms or endoscopic alterations occur. Therefore, FMT might be an important tool for repetitive and non-invasive monitoring of inflammatory cell infiltrate in intestinal inflammation.

## 1. Introduction

Inflammatory bowel diseases (IBD) are chronic relapsing and remitting inflammatory disorders of the gastrointestinal tract which affect an increasing number of patients especially in industrialized countries with current prevalence rates for Crohn’s disease (CD) and ulcerative colitis (UC) exceeding 200–250/100,000 [1,2]. Although the pathogenesis of IBD is still incompletely understood, recruitment, activation, and infiltration of immune cells represent crucial steps in the pathogenesis of IBD [3]. Therefore, inhibition of gut homing of immune cells is a promising therapeutic option. While some antibodies inhibiting integrin-dependent leucocyte migration are already available for patients [4,5], other substances have yielded promising results in preclinical tests [6,7,8,9,10].

Neutrophils are often the first immune cells recruited to the site of inflammation and play a key role in combatting microbial invasion [11]. When these mechanisms become uncontrolled, extensive tissue damage and development of chronic disease might be the consequence. The role of neutrophils in the pathogenesis of IBD remains controversial and possibly differs between UC and CD and no therapeutic strategies exist to selectively target neutrophils [12,13]. Monitoring neutrophil trafficking to the site of inflammation would be helpful for understanding their contribution to the pathogenesis of disease and furthermore, this would help to visualize and assess the anti-inflammatory effects of new drugs.

In the preclinical setting, Dextran sodium sulfate (DSS)-induced colitis is a standard chemically induced mouse model of intestinal inflammation resembling IBD and proved useful for examining underlying mechanisms of inflammatory pathology associated with IBD with focus on the involvement of the innate immune system [14]. However, common clinical readouts mainly rely on surrogate parameters of inflammation, endoscopic assessment is an invasive procedure and post- mortem analyses such as immunohistochemistry prevent longitudinal analyses at repetitive time points.

Fluorescence mediated tomography (FMT) could overcome this limitation, enabling non-invasive and intraindividual follow-up examinations, which might also result in a decrease of research-animals needed for a study [15,16]. While several different optical imaging techniques exist for in vivo fluorescence imaging, only recently tomographic systems allowing quantitative three-dimensional tissue analysis have been developed with the advent of sensitive detectors and monochromatic light sources as well as fluorescent probes which emit light in the near-infrared spectrum, where surrounding tissue offers low absorption [17,18]. As opposed to conventional imaging techniques, such as endoscopy or cross-sectioning imaging modalities (e.g., computed tomography (CT), magnetic resonance imaging (MRI), or ultrasound (US)) which rely mostly on physical parameters to visualize morphology, optical imaging can provide additional information on underlying (patho-)physiological processes using endogenous or exogenous fluorescent probes [19], such as fluorescence labeled specific antibodies against Gr-1, which is a common antigen expressed on neutrophil granulocytes [20].

The aim of this study was to evaluate Gr-1 targeted FMT for detection and quantification of neutrophil infiltration in a murine model of intestinal inflammation and to visualize neutrophil recruitment and the kinetics of infiltration at repetitive time points in the development of experimental colitis.

## 2. Materials and Methods

### 2.1. Animals and Colitis Induction

Female wildtype C57BL/6 mice were purchased from Charles River Laboratories (Sulzfeld, Germany) and were group-housed (maximum five per cage) at the animal facility of the University Hospital of Münster in a temperature-controlled room at 22–24 °C with 12 h light/dark cycle under pathogen-free conditions with free access to a standard rodent chow diet and tap water until reaching the desired weight (20–25 g at 6–9 weeks of age). DSS colitis was induced as described elsewhere [14]. Briefly, mice were administered 2.5% (w/v) DSS (MW~40,000) in drinking water for seven consecutive days. Non-colitic control mice received drinking water without DSS. Animals were monitored daily throughout the entire experiment and euthanized 10 days after colitis induction. Euthanasia was performed via CO_2_ asphyxia, followed by rapid cervical dislocation. The study design and all experiments have been approved according the German Animal Protection Lwa (Tierschutzgeset) by the regional federal government authority (Landesamt für Natur, Umwelt und Verbraucherschutz [LANUV]) Nordrhein-Westfalen under the reference AZ84-02.04.2013.A093 (Date of approval 14 March 2017).

### 2.2. Assessment of Colitis

The course of inflammation was monitored by daily measurements of weight loss and presence of blood in the stools using a guaiac paper test (Roche Diagnostics, Mannheim, Germany). Animals were euthanized 10 days after colitis induction and colons were removed, measured, and subjected to ex vivo fluorescence imaging. The colon was then opened longitudinally. A fragment from the distal colon was obtained, immediately placed in a 1.5-mL cryogenic tube and frozen in liquid nitrogen, then stored at −70 °C until further use (e.g., myeloperoxidase (MPO) measurements, cytokine measurements). The remaining colon was rolled up longitudinally with the mucosa outwards and embedded as “Swiss rolls” in a fixative (Optimal cutting temperature formulation, O.C.T. compound from Tissue-Tek, Sakura Finetek, Zoeterwoude, the Netherlands) and frozen at −80 °C. For histological analysis, cryostat sections from proximal, medial, and distal colon were picked up, stained with hematoxylin and eosin and graded in a blinded fashion using a colitis score, as previously described by Dieleman et al. [21]. Briefly, all sections were graded with respect to the amount of inflammation (none, slight, moderate, severe), extent of injury (none, mucosal, mucosal and submucosal, transmural) and crypt damage (none, basal 1/3 damaged, basal 2/3 damaged, only surface epithelium intact, entire crypt, and epithelium lost). These changes were also quantified as to the percentage involvement by the disease process: (1) 1–25%; (2) 26–50%; (3) 51–75%; (4) 76–100%. Each section was then scored for each feature separately by establishing the product of the grade for that feature and the percentage involvement.

Immunohistochemistry was performed as described previously [16,22]. Briefly, 4 µm acetone fixed frozen sections were blocked in 5% rat serum and incubated overnight at 4 °C with the diluted biotinylated primary antibody (1:500, rat-anti-mouse Gr-1-granulocyte (myeloid)-differentiation antigen Gr-1 (Ly6G)-BD Pharmingen, Heidelberg Germany). Sections were washed thrice in tris-buffered saline (TBS) and incubated with Streptavidin-Alexa546 (Molecular Probes, Darmstadt, Germany) in PBS/bovine serum albumin (BSA) (0.1% w/v) for 1 h at room temperature. Sections were washed three times and contrast staining was performed using DAPI (1:1000, Sigma-Aldrich, Munich, Germany). Fluorescence images were acquired using a Zeiss LSM510 confocal microscope. The numbers of Gr-1 positive cells in the colon was counted per high power field (HPF) in three sections of the proximal, intermediate, and distal colon.

### 2.3. Tissue Myeloperoxidase (MPO) Assay

For measurements of MPO levels, colon samples were rinsed with phosphate buffered saline (PBS), blotted dry, and snap-frozen for further use. Thawed samples were weighed, homogenized in ready to use sample buffer, sonicated, and centrifuged (200× *g*, 10 min, 4 °C). MPO levels in equal amounts of the homogenate were measured using a commercially available enzyme-linked immuno-sorbent assay (ELISA) kit (Immundiagnostik AG, Bensheim, Germany) according to the manufacturer’s instructions. MPO levels were then normalized by total protein concentration and expressed as ng/mg protein.

### 2.4. Real Time (RT)-PCR Analysis

Total RNA was isolated from freeze-thawed colon tissue using Trizol (Invitrogen, La Jolla, CA, USA) and subjected to mRNA purification using lithium chloride precipitation. Reverse-transcription to cDNA was performed using Invitrogen (Carlsbad, CA, USA) cDNA synthesis kit according to the manufacturer’s instructions. The real-time PCR reaction was performed on the ABI Prism 7000 instrument with SYBR Green PCR master mix (Qiagen, Germantown, MD, USA) with an initial denaturation step at 95 °C for 15 min, followed by 45 cycles of 94 °C for 15 s annealing temperature with extension step for 30 s at 55 °C by using respective following primers of various target genes. Mouse mRNA primers were purchased from Qiagen (Germantown, MD, USA) (TNF-α: NM_013693; IL-1β: NM_008361; CXCL2: NM_009140). The relative changes in TNF-α, IL-1β, and CXCL-2 with respect to GAPDH expression were examined using the 2−ΔΔCt method with healthy mice as reference. All cDNA samples were analyzed in triplicates.

### 2.5. Fluorescence-Mediated Tomography

FMT in mice has been described previously [16]. For Gr-1 targeted FMT, a monoclonal purified rat-anti mouse Gr-1 (rat IgG2b) antibody (BioLegend, London, UK) was labelled with Cy5.5-NHS-ester (excitation 630 nm; emission 680 nm) for optical imaging approaches. Purified antibody was solved in NaHCO_3_ buffer (0.1 mmol/L, pH 8.3) and incubated with Cy5.5-NHS-ester for 1 h. The labelled antibody was purified from unbound precursors by chromatography and resolved in PBS for in vivo application. The efficacy of labelling (dye/antibody ratio) was determined based on ultraviolet-spectra of the purified dye–antibody compound using PBS as a reference buffer. Typically, the labelling resulted in 2.5–3.0 fluorochrome molecules per antibody. Mice were injected with the fluorescence-labelled antibody 24 h prior to subjecting the animals to FMT scanning. Mice received the labelled antibody in the amount corresponding to 2.0 nmoL Cy5.5 (80 µL antibody solution) intravenously via the tail vein. Control mice received an equally labelled unspecific control antibody (purified rat IgG2b, κ Isotype control; BioLegend, London, UK). All in vivo imaging experiments were performed at a VisEN 2500 Beta FMT device (VisEn Medical, Woburn, MAS, USA) for small animal fluorescence imaging. The device was equipped with a Cy5.5-adapted filter set for excitation and emission. Animals were held under isoflurane inhalation anesthesia (0.3 l O_2_/min; 1.5–2.0% isoflurane) for the time of examination (about 10 min for complete scan) to prevent movement artefacts and additional stress. The imaging data were screened for fluorescence uptake in the abdominal region and fluorescence signal was recorded and quantified for the whole abdominal region as well as for distinct segments of the bowel by manual placing of regions of interest (ROI) on the 3D maps. Data were documented as total amounts of fluorescence signal in the ROI.

### 2.6. Endoscopy

Murine endoscopy and grading of inflammatory changes were performed as previously described [15,23]. Briefly, endoscopy was performed in vivo prior to FMT scanning using a rigid murine colonoscope with a diameter of 1.9 mm and 10 cm length (Coloview Endoscopic System^®^, KARL STORZ GmbH & Co. KG, Tuttlingen, Germany). Examinations were performed under sedation with isoflurane (0.3 l O_2_/min; 1.5–2.0% isoflurane). To quantify the inflammation induced mucosal damage, the murine endoscopic index of colitis severity (MEICS) was used, which consists of five parameters to assess inflammation: Thickening of the colon wall, changes of the vascular pattern, presence of fibrin, granularity of the mucosal surface, and stool consistence [24].

### 2.7. Preparation of Leukocytes and Flow Cytometry

Preparation of leukocytes and flow cytometry has been described previously [25]. Briefly, blood was obtained from anesthetized animals prior to endoscopy and scanning by retroorbital punction and leukocytes were isolated using Biocoll according to the manufacturer’s instructions and cells (1 × 10^6^ /mL) were incubated in fluorescence-activated cell sorting (FACS) buffer (BD Pharmingen, Heidelberg, Germany) with anti-CD11b or anti-Ly6C antibodies (1:100 *v/v* each) for 30 min at 4 °C. Anti-CD11b (rat-anti-mouse, TC conjugated) was from Caltec (San Francisco, CA, USA). Anti Ly6C (rat-anti-mouse, FITC conjugated) was from BD Pharmingen (Heidelberg, Germany). Cells were analyzed by flow cytometry (FACSCalibur, BD Biosciences GmbH, Heidelberg, Germany). Each measurement contained a defined number of 2 × 10^5^ cells.

### 2.8. Experimental Design and Statistical Analysis

Endoscopy and FMT as well as FACS analysis were performed before (day 0) as well as on day 4 and 10 (end of the experiment) after colitis induction in mice, which were injected with either the fluorescence labeled specific or the unspecific control antibody. Procedures performed before colitis induction (day 0) served as pre-experiment control and colitis induced alterations were monitored intra-individually. Additionally, healthy mice (receiving no DSS) served as controls for monitoring weight-loss and fecal occult blood throughout the experiment as well as post-mortem analyses. Experiments were performed in groups of five animals each. Each outcome was confirmed in two experiments.

Statistical analysis was performed with SigmaPlot version 11 (Systat Software Inc., San Jose, CA USA) using Mann-Whitney-U test and ANOVA. Post-hoc comparisons were conducted with the Student–Newman–Keuls (S.N.K) test, if F achieved *p* < 0.05 and there was no significant variance inhomogeneity. In the case of a significant variance inhomogeneity (*p* < 0.05 in the Levene test), Welch’s test was used followed by Games-Howell post hoc test for multiple comparisons, if F achieved a *p* < 0.05. All values are reported as mean ± SE. Statistical significance was set at *p* < 0.05 (as indicated in the figures).

## 3. Results

### 3.1. General Assessment of Colitis

**Figure 1:**
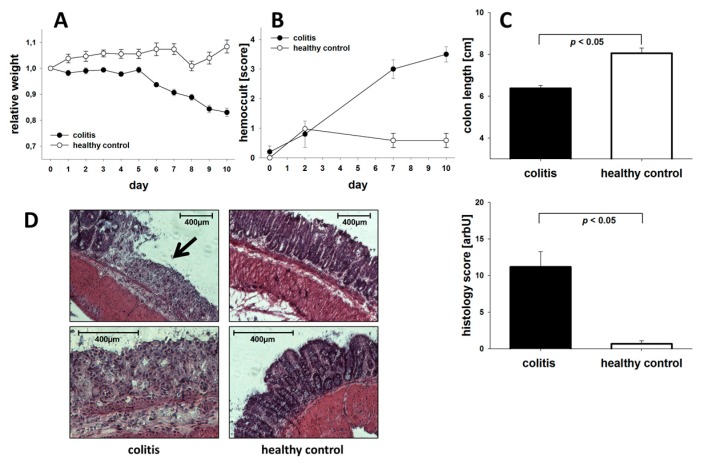
Assessment of clinical course and intestinal histological damage of acute Dextran sodium sulfate (DSS)- colitis. DSS colitis was induced in C57BL/6 mice by application of DSS (2.5% w/v) in drinking water for seven days. Control mice received drinking water alone. Animals were euthanized at day 10. Shown are data from one experiment (n = 5 per group). Results were confirmed in two experiments. (**A**) Changes in body weight are shown relative to the initial weight. Data were analyzed using ANOVA followed by Student–Neuman–Keul (S.N.K.) post hoc test. (**B**) Fecal blood excretion as determined by guaiac paper test (hemoccult, 0 = negative, 4 = blood macroscopically). Data analysis by ANOVA and S.N.K. post hoc test. (**C**) Post-mortem colon length. (**D**) Histological damage as determined by degree of crypt destruction and extent of inflammation. Shown are representative histological images (haematoxylin/eosin staining; magnifications ×10 of colitic mice or control). Bar graph: Injury score.

DSS-induced colitis resembles human ulcerative colitis with respect to weight loss, rectal bleeding, and mucosal damage [14]. For this study, decreasing body weight and fecal occult blood were used as clinical indices of inflammation. As shown in Figure 1A, application of DSS led to a decrease of body weight in all animals beginning on day five, while animals receiving water alone showed no significant change of body weight. Additionally, colitic animals showed increased daily excretion of fecal occult blood (Figure 1B). Colonic shortening is considered a macroscopic indicator of inflammation-induced fibrosis in the course of colitis and confirmatory, colitic animals had significantly shorter colons in post-mortem measurements of colon length as compared to healthy control mice (Figure 1C). For a direct assessment of colonic damage, post-mortem blinded histological analysis of H&E stained colon sections was performed on day 10 after DSS application. Figure 1D (left image) demonstrates inflammatory changes typical for DSS-induced colitis (loss of goblet cells, crypt damage, mucosal ulceration, and accompanying submucosal edema). As expected, healthy control mice displayed intact mucosa (Figure 1D right image). Quantitative assessment of histological damage using an injury score based on the degree and extent of inflammation, crypt damage, and percent involvement (Dieleman Score [21]) revealed a high degree of inflammation and histological damage in colitic mice (Figure 1D, bar graph).

### 3.2. Post-Mortem Assessment of Neutrophil Recruitment

**Figure 2:**
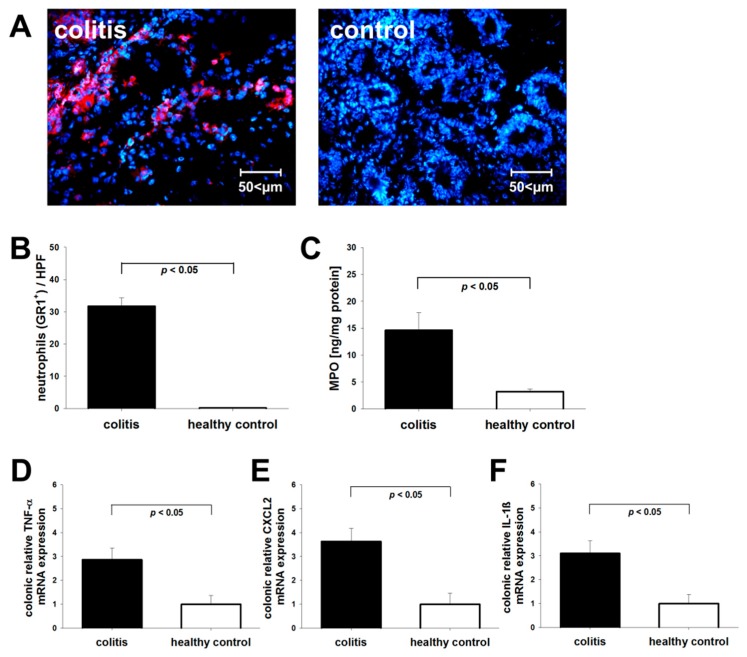
Assessment of intestinal neutrophil infiltrate and pro-inflammatory cytokines. Infiltration of neutrophils into the intestinal wall was determined post-mortem in DSS-colitic mice and healthy controls. Shown are data from one experiment (n = 5 per group). Results were confirmed in two experiments. (**A**) Immunofluorescent visualization of neutrophil infiltration in the mucosa. Shown are representative images of anti-Gr-1 staining in colitic and control mice. (**B**) Bar graph: Cell count/high power field (HPF). (**C**) Confirmatory measurements of myeloperoxidase (MPO) concentration in colon tissue of colitic and non-colitic control mice. mRNA levels of pro-inflammatory cytokines TNF-α (**D**), CXCL2, (**E**) and IL-1β (**F**). mRNA was isolated from the colon of mice for the RTq-PCR analysis. The cytokine mRNA expression level in the healthy control group was set as 100%, and mRNA expression levels in colitic mice were compared with the control group.

Infiltration of neutrophils into the intestinal wall of colitic mice was quantified post-mortem ten days after induction of colitis by immunofluorescence staining for the neutrophil marker Gr-1. Compared to non-colitic control mice, colitic animals showed a significantly elevated presence of Gr-1 positive neutrophils in the intestinal wall (Figure 2B). Confirmatory, colonic MPO levels, correlating with the number of neutrophils and the severity of inflammatory activity, were significantly increased in colon tissue obtained from colitic mice versus non-colitic animals (Figure 2C). Additionally, mRNA levels of pro-inflammatory cytokines were significantly elevated in inflamed colonic tissue compared with healthy control animals (Figure 2D–F).

### 3.3. Dss-Colitis Induces Systemic Changes in Macrophage Subpopulations

**Figure 3:**
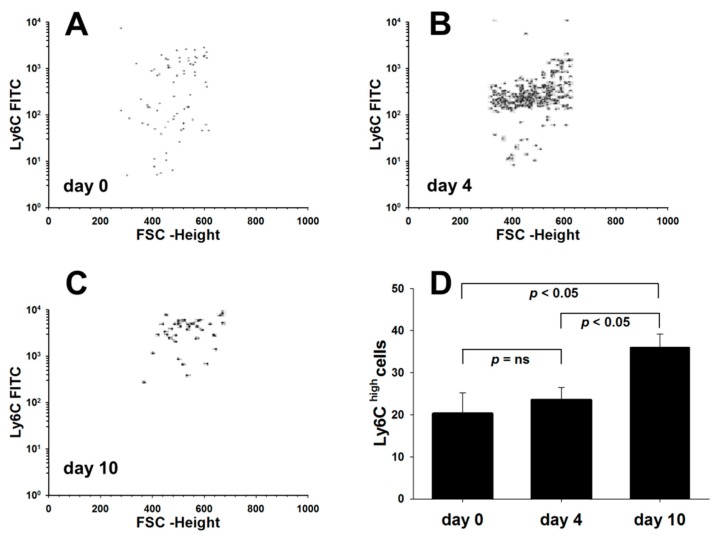
Inflammation induced changes in the expression of blood monocytes during the course of colitis. Murine peripheral blood monocytes were analyzed by flow cytometry for the expression of Ly-6C on CD11b-positive cells. Shown are data from one experiment (n = 5 per group), confirmed in two experiments. Fluorescence-activated cell sorting (FACS) dot plots of Ly6C expression on SSC^low^CD11b^high^ cells of mice before (**A**; day 0), during (**B**; day 4), and after colitis induction (**C**; day 10). (**D**) The number of Ly6C^hi^ monocytes circulating in mice prior (day 0) and after colitis induction (day 4 and 10) per 2 × 10^5^ cells measured.

Several subpopulations of murine blood monocytes, which differ in their capacity to become recruited to inflammatory sites and to differentiate into pro-inflammatory cells, have been described. Thus, we additionally quantified blood monocytes according to the level of CD11b and Ly6C expressions. As shown in Figure 3, the induction of DSS-colitis in mice produced a distinct shift towards a higher proportion of inflammatory CD11b^high^Ly6C^high^ monocytes as compared with pre-experiment conditions.

### 3.4. Fluorescence Mediated Tomography Scan Detects Early Neutrophil Infiltration in Murine Colitis

**Figure 4:**
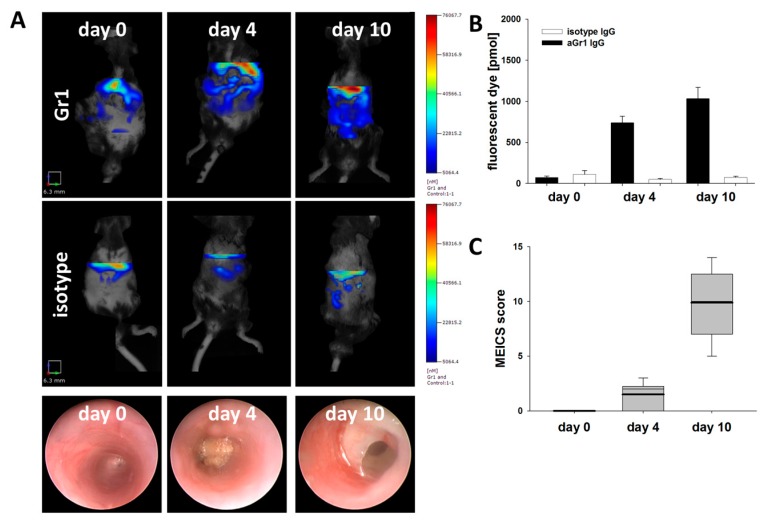
Fluorescence-Mediated Tomography (FMT) visualization of neutrophil infiltrate in murine colitis and correlating white light endoscopy. FMT scans in mice prior (day 0), during (day 4), and after (day 10) induction of colitis. Mice were injected with Cy 5.5 conjugated antibody against murine Gr-1 or equally labeled unspecific control (rat IgG). Prior to each scan, mice were subjected to endoscopy to assess mucosal inflammation of the bowel. Shown are data from one experiment (n = 5 per group) and results were confirmed in two experiments. (**A**) Representative images of FMT scans are shown with color-coded fluorescence intensity corresponding to the extent of inflammatory infiltrate in mice injected with the specific probe (upper panels) and unspecific control (middle panels). In both cases, a region of interest (ROI) of equal size was placed transverse in the upper abdomen for further analysis. Corresponding white-light endoscopic images from the respective colitic mice for each time point acquired prior to FMT scanning are shown in the bottom panels. (**B**) Total fluorescence intensity in pmol dye was determined for all mice in ROI at three time points and depicted ± SEM. FMT-data were analyzed using ANOVA followed by Student–Neuman–Keul (S.N.K.) post hoc test. (**C**) Endoscopic mucosal damage as assessed by murine endoscopic index of colitis severity (MEICS) score was determined prior to each FMT scan and depicted ± SEM. Statistical significance was calculated by Mann–Whitney U test.

For direct visualization of neutrophil recruitment and infiltration into the murine intestine, FMT was employed using a fluorescence labelled antibody against murine Gr-1. Tracer accumulation was documented and quantified as total amounts of fluorescence signal in the ROI. In parallel, the degree of colonic inflammation was assessed endoscopically. Before induction of colitis, FMT detected no significant tracer uptake in the intestinal wall of mice (Figure 4A left panel and Figure 4B). As early as four days after DSS application, FMT showed a significant increase in the level of fluorescence as compared to pre-experimental conditions, while neither standard clinical parameters such as loss of body weight (Figure 1A) nor conventional endoscopy showed relevant signs of inflammation (Figure 4A middle panel and Figure 4C), indicative of an early infiltration of inflammatory neutrophils, which conventional parameters failed to detect. Highly sensitive measurements of fecal occult blood excretion detected a minimal increase in fecal occult blood early after DSS exposure, suggestive of mucosal injury (Figure 1B). Confirmatory, a shift towards proinflammatory CD11b^high^Ly6C^high^ monocytes was detected in peripheral blood samples at this time (Figure 3). Full-fledged inflammation with significantly increased neutrophil infiltration as compared to pre-experimental conditions and day four after colitis induction was measured by FMT at day 10 with corresponding endoscopically observed macroscopic colonic inflammation (Figure 4A right panel and Figure 4B,C). Mice injected with an equally labelled unspecific isotype control IgG antibody showed no increased tracer accumulation throughout the experiment (Figure 4A middle panels, isotype control).

### 3.5. Specificity of FMT Detected Gr-1 Tracer Signal

**Figure 5:**
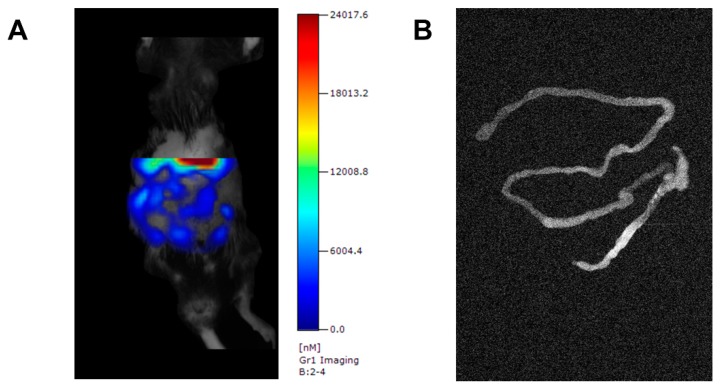
Ex vivo validation of tracer specificity. To verify the colonic origin of the tracer signal, in vivo FMT scans in colitic mice injected with the specific probe (anti mouse Gr-1) were followed by ex vivo planar fluorescence scans of explanted intestines (n = 5). Representative images are shown depicting in vivo FMT scans of colitic mice with color-coded fluorescence intensity corresponding to the extent of inflammatory infiltrate (**A**) and subsequent ex vivo fluorescence scans (**B**) with fluorescence intensity (brightness) corresponding to the extent of inflammatory infiltrate.

To further validate the specificity of the probe-target interactions, ex vivo measurements of Gr-1 directed tracer accumulation were performed on freshly explanted colons. Representative FMT images of the abdominal region and explanted colons are shown in Figure 5, verifying the colonic origin of the in vivo detected Gr-1 specific signal.

## 4. Discussion

The pathogenesis of IBD is still incompletely understood; however, infiltration and activation of various immune cells in the gut wall are crucial steps in the development of intestinal inflammation. Generally, the recruitment of inflammatory cells to the site of infection or inflammation represents an important step in many immune-mediated diseases, such as autoimmune disease, infection, vascular disease, or tumorigenesis. The understanding of these processes provides valuable insights into the underlying pathophysiological mechanisms and enables promising novel therapeutic approaches to more selectively target intestinal inflammation [26,27,28]. Recently, anti-adhesion therapies have been successfully employed in clinical trials to treat IBD and have been approved for patient care [29]. Second-generation anti-adhesion therapies that more selectively target immune cell trafficking into the inflamed intestine have also been introduced [4,5]. Additionally, further anti-trafficking molecules have been developed, which selectively interfere with immune cell recirculation and infiltration. [6,30].

The contribution of neutrophils to the development of IBD remains controversial and might differ between UC and CD [13]. In detail, for UC some studies suggest that uncontrolled neutrophil activation leads to tissue damage and development of chronic disease due to impaired control-feedback mechanisms [31,32]. In CD impaired neutrophil function might result in an insufficient control of microbial invasion and a subsequent uncontrolled inflammatory reaction [33,34]. Interestingly, IBD-like colitis has been described in chronic granulomatous diseases and glycogen storage disease-type 1b. Neutropenia and defective neutrophil functions may account for the condition, although the precise mechanism remains elusive [35,36]. It is speculated that neutrophil deficiency leads to chronic low-grade infections of the gut, which may increase the predisposition to intestinal inflammation [37]. Our findings of early neutrophil infiltration after DSS-induced mucosal injury might support this notion. However, the role of neutrophils in the resolution of inflammation remains to be elucidated [13]. Although neutrophils are the first immune cells recruited to the site of inflammation, their activity needs to be tightly regulated to limit collateral damage to the tissue and avoid evolution toward chronic diseases.

At present, data on the role of neutrophils in DSS-colitis are conflicting. While some studies showed beneficial effects with ameliorated colitis after neutrophil depletion [38], others have demonstrated disease exacerbation after depletion [39]. The role of neutrophils in intestinal inflammation might be dual. The acute inflammatory response is needed for bacterial clearance after epithelial barrier disruption, while excessive or prolonged neutrophil activation can lead to chronic inflammation. No unequivocal conclusion can be drawn based on the present results as to which role neutrophils play in the resolution or perpetuation of prolonged inflammation. However, the present study provides several pieces of evidence pointing to the importance and function of neutrophils in acute intestinal inflammation and possibly bacterial clearance. While mice responded with clinical signs of inflammation (weight loss, severe excretion of blood with feces) 5–6 days after DSS administration, FMT showed an infiltration of neutrophils relevantly earlier, also before morphologic inflammatory changes could be observed by endoscopy. This was accompanied by an inflammatory left shift in the blood towards CD11b^+^/Ly6c^high^ monocytes, which constitute a subpopulation of aggressive pro-inflammatory monocytes that home to inflammation sites and give rise to activated macrophages [25]. Additionally, the concentration of MPO, which reflects both, neutrophil numbers and inflammatory activity, was greatly elevated in colonic tissue. MPO is contained in primary granules formed early during neutrophil maturation and is liberated in copious amounts by activated neutrophils to amplify the inflammatory response by inducing oxidative modification of proteins and lipids [40]. Moreover, the concentration of pro-inflammatory cytokines associated with neutrophil recruitment and activation was greatly elevated in colon samples obtained from colitic mice.

Although the exact mechanisms of colitis induction by DSS are only incompletely understood, it is one of the most widely used models of IBD due to its rapidity, uniformity, and reproducibility of colonic lesions and well documented involvement of the innate immune system. DSS causes a disruption of the colonic epithelial barrier with increased permeability for luminal antigens and microorganisms [41]. Standard clinical surrogate parameters for disease severity as well as morphological changes were observed after DSS application in our model (weight loss, fecal occult blood secretion, histological damage to the large bowel, colonic shortening). Yet, mice can display differential susceptibilities to colitis due to genetic (strain, gender) and microbiologic variations (intestinal flora) [42,43]. To ensure comparable results, only cohoused female mice of the same origin and weight were used in this study, although preliminary experiments with male mice have shown similar clinical responses to DSS exposure (data not shown). While DSS colitis also develops in the absence of T cells mediating adaptive immunity, T cell-mediated models of intestinal inflammation in mice that also mimic the human conditions have been described [41,44]. Further studies will be needed to elucidate the role of neutrophils in other models of inflammation, especially with respect to prolonged neutrophil activation which can lead to chronic inflammation. Elucidating these pathways could provide new therapeutic opportunities to modulate neutrophil migration without eliminating it, therefore preserving host-defense. Our Gr-1-targeted scan could aid in clarifying the role of neutrophils in IBD as it can monitor neutrophil infiltration at a very early time point in the development of inflammation even before clinical symptoms or endoscopic alterations occur, works non-invasively and additionally it allows the acquisition of repetitive imaging series over time.

Despite recent advances in medical imaging techniques, the detection of inflammatory processes or tumors in their earliest stages of development remains challenging. While conventional imaging modalities rely on physical or physiological parameters, molecular imaging enables a visualization of specific molecular markers in vivo [45]. However, most systems are limited to rapid planar imaging of superficial lesions, since signals emitted from deeper layers of tissue evade planar imaging due to light absorption and scattering [46,47]. FMT can overcome this limitation, because the imaging signal is corrected for scattering and light absorption and data acquisition in a multi-projection way enables the calculation of a virtual 3D dataset [19]. The underlying mathematical algorithms enable a quantification of the tracer concentration in the ROI [47]. Moreover, the non-invasive nature of the technique allows repetitive examinations of the same animals at various time points of the experiments. The results of the FMT scan indicate the development of the increasing neutrophil infiltration over time in individual mice. This does not only enable a unique longitudinal experimental design but might also decrease the number of animals needed as opposed to post-mortem measurements at different time points, which might be desirable from an animal ethics point of view.

Poor spatial resolution as compared to anatomical imaging techniques might be a relevant limitation of FMT, since this can compromise the allocation of molecular information to specific anatomic structures, depending on the desired target. However, ex vivo scanning clearly revealed the colonic origin of the in vivo acquired fluorescence signal, indicating the sufficient spatial resolution of the used scan. Restricted penetration depth of light into tissue, which requires the use of probes absorbing and fluorescing in the red to near infrared (NIR) spectral range is another limitation of FMT, which therefore can only be applied in an experimental pre-clinical setting of small animal imaging [47]. Recently, optoacoustic imaging has been introduced, combining the advantages of morphologic US and the possibility to obtain molecular information from fluorescence as a potential translational application [48].

A wide array of probe target combinations has been established and molecular imaging, as presented in this study, can be used in many settings to monitor cell trafficking, tumor growth, invasion, and metastasis as well as other immunological processes. For deep tissue imaging, we employed a dye operating in the near-infrared spectrum (e.g., Cy5.5, λex/em 680/720 nm) that had previously been proven to be optimal for monitoring intestinal inflammation in mice [16]. As little is known about recirculation times of Gr-1 molecules on neutrophils, we cannot fully exclude the possibility of tracer accumulation over the time of the experiment when used at repetitive time points. Since some studies have suggested that the anti-Gr1 antibody employed in this study can reside on cells for up to four days in vivo, scans were performed at least four days apart [49]. Scanning of mice previously to secondary injections of tracer provided no evidence for accumulation and served as baseline for the subsequent scan (data not shown). The optimal time point between tracer application and the imaging procedure was determined previously [6,16], but might vary according to the chosen probe-target combination. For example, antibody targeting of tumor receptors might be influenced by the diffusion rate to tumors, uptake, and metabolism [50].

Taken together, molecular FMT enables repetitive monitoring of the disease course and underlying pathophysiological processes in an experimental model of colitis and visualizes early inflammatory processes which conventional readouts or endoscopy fail to detect at such an early time point. It can be applied in various other biomedical small animal studies and might be useful to accelerate drug testing or as an objective endpoint in individualized therapies. Therefore, FMT-targeting of Gr-1 expressing neutrophils might be an important visualization tool for repetitive and non-invasive monitoring of inflammatory cell infiltrate in intestinal inflammation.
